# Between-Class Adversarial Training for Improving Adversarial Robustness of Image Classification

**DOI:** 10.3390/s23063252

**Published:** 2023-03-20

**Authors:** Desheng Wang, Weidong Jin, Yunpu Wu

**Affiliations:** 1 School of Electrical Engineering, Southwest Jiaotong University, Chengdu 611756, China; 2 China-ASEAN International Joint Laboratory of Integrated Transportation, Nanning University, Nanning 541699, China; 3 School of Electrical Engineering and Electronic Information, Xihua University, Chengdu 610039, China

**Keywords:** adversarial training, between-class learning, robustness, regularization

## Abstract

Deep neural networks (DNNs) have been known to be vulnerable to adversarial attacks. Adversarial training (AT) is, so far, the only method that can guarantee the robustness of DNNs to adversarial attacks. However, the robustness generalization accuracy gain of AT is still far lower than the standard generalization accuracy of an undefended model, and there is known to be a trade-off between the standard generalization accuracy and the robustness generalization accuracy of an adversarially trained model. In order to improve the robustness generalization and the standard generalization performance trade-off of AT, we propose a novel defense algorithm called Between-Class Adversarial Training (BCAT) that combines Between-Class learning (BC-learning) with standard AT. Specifically, BCAT mixes two adversarial examples from different classes and uses the mixed between-class adversarial examples to train a model instead of original adversarial examples during AT. We further propose BCAT+ which adopts a more powerful mixing method. BCAT and BCAT+ impose effective regularization on the feature distribution of adversarial examples to enlarge between-class distance, thus improving the robustness generalization and the standard generalization performance of AT. The proposed algorithms do not introduce any hyperparameters into standard AT; therefore, the process of hyperparameters searching can be avoided. We evaluate the proposed algorithms under both white-box attacks and black-box attacks using a spectrum of perturbation values on CIFAR-10, CIFAR-100, and SVHN datasets. The research findings indicate that our algorithms achieve better global robustness generalization performance than the state-of-the-art adversarial defense methods.

## 1. Introduction

DNNs have achieved impressive success in many computer vision tasks such as image classification [1], object detection [2], and semantic segmentation [3]. However, recent studies on adversarial examples [4,5] reveal the weakness of DNNs on robustness, showing that carefully designed small perturbations can mislead a network to produce incorrect outputs with high confidence. In the context of image classification, the perturbations in adversarial examples are human-imperceptible and can change the prediction of a classification model to incorrect classes. Moreover, adversarial examples can also transfer across different model parameters and even architectures. As a result, adversarial examples become a significant threat to deep learning-based security-crucial applications such as self-driving cars [6], person detection systems [7], or medical diagnosis systems [8]; hence, it is a crucial issue to develop methods that improve the robustness of DNNs against adversarial examples.

Methods that generate adversarial examples are called adversarial attacks [4,5,9,10,11,12,13,14,15,16]. Take image classification as an example, the adversarial attacks methods generate adversarial examples by adding carefully designed $L_{p}$ norm bounded perturbations to clean examples. According to the type of the norm, adversarial examples can be categorized into $L_{0}$ norm-based ones [11,12], $L_{1}$ norm-based ones [15], $L_{2}$ norm-based ones [13,14], and $L_{\infty }$ norm-based ones [4,5,9]. Moreover, adversarial attacks can have full access to model architecture, parameters, training algorithm, and training dataset, which is called white-box attacks [4,5,9,10,11,12,13,14,15], or can only query and observe outputs, which is called black-box attacks [16]. Furthermore, adversarial attacks can be targeted or untargeted. Targeted attacks make a model output a chosen class, different from the ground-truth class; untargeted attacks make a model output any class, different from the ground-truth class.

Adversarial attacks have attracted considerable research interest in developing adversarial defense to improve the adversarial robustness of DNNs. For example, feature squeezing [17] reduces the power of the adversary by reducing the color bit depth of pixel values of input images and spatial smoothing. Stochastic Activation Pruning [18] and Deep Contractive Network [19] modify the network architecture to improve the adversarial robustness of DNNs. Defense-gan [20], Pixeldefend [21], and Magnet [22] add auxiliary networks to make DNNs robust to adversarial examples. Nevertheless, these defense methods proposed are demonstrated to give a false sense of robustness due to obfuscated gradients or evaluated under weak threat models [23,24]. It is generally accepted that AT [4,5,9] that trains DNNs with adversarial examples is, so far, the only method that can improve the robustness of DNNs against adversarial examples. However, AT is known to damage the accuracy on clean examples [4,5,9]; in addition, the adversarial generalization performance gained from AT is much lower than the standard generalization performance gained from standard training. Schmidt et al. [25] demonstrate that this is due to the higher sample complexity needed by robustness generalization than standard generalization.

Improving the robustness of DNNs against adversarial examples can be viewed as the problem of reducing overfitting, namely improving the generalization performance of DNNs on testing adversarial examples. Regularization is a commonly used method to reduce overfitting and improve the standard generalization of DNNs. Well-known regularization methods include weight decay [26], dropout [27], and data augmentation [28]. Weight decay regularizes the DNNs on the model side by introducing a regularization term into the loss function to penalize high weight values, which prevents the model from getting too complex, thus reducing overfitting and improving generalization. Dropout also works on the model side by randomly dropping out nodes during training which approximates assembling a large number of models with different architectures, thus reducing overfitting and improving generalization. Different from weight decay and dropout, data augmentation regularizes the DNNs on the data side. By applying geometric transformations such as flipping, cropping, rotation, and translation to already existing data [28] or generating synthetic data [29], data augmentation increases sample complexity, thus reducing overfitting and improving generalization. BC-learning [30,31] is a recently proposed data augmentation method that mixes two examples belonging to different classes with a random ratio to generate between-class examples, then inputs the mixed between-class examples to a model and trains the model to output the mixing ratio. BC-learning imposes regularization on the feature distribution of clean examples, which enlarges the between-class distance. BC-learning was originally designed for sound recognition [30,31], but was then found to also improve the standard generalization of image classification [30,31]. However, little work has been done to study the effectiveness of BC-learning on the robustness generalization of image classification.

This paper aims to answer the question of whether BC-learning can further improve the robustness generalization of adversarially trained DNNs on the image classification task by regularizing the feature distribution of adversarial examples. We first introduce an intriguing property of adversarial examples called Label-Feature Distribution Mismatch and point out that the Label-Feature Distribution Mismatch property is a reason that causes poor generalization performance of DNNs against adversarial examples. We then propose a novel adversarial training algorithm named BCAT that combines BC-learning with AT. Specifically, BCAT trains DNNs on between-class adversarial examples mixed with two adversarial examples belonging to different classes to output the mixing ratio. We further propose BCAT+ which adopts a more powerful mixing method. Experimental results demonstrate that BCAT and BCAT+ can effectively regularize the feature distribution of adversarial examples to enlarge the between-class distance. Models trained using BCAT and BCAT+ achieve better global adversarial robustness generalization performance than the state-of-the-art adversarial defense methods on CIFAR-10, CIFAR-100, and SVHN datasets. The main contributions of this paper are summarized below:

- We introduce the Label-Feature Distribution Mismatch property of adversarial examples and point out that the Label-Feature Distribution Mismatch property is a reason that causes poor adversarial robustness generalization performance of DNNs against adversarial examples;
- We propose two novel adversarial defense algorithms named BCAT and BCAT+ that train DNNs to output the mixing ratio of two adversarial examples with different real labels, which impose effective regularization on the feature distribution of adversarial examples;
- We design extensive experiments to evaluate the proposed BCAT and BCAT+ algorithms under both white-box and black-box attacks on CIFAR-10, CIFAR-100, and SVHN datasets. The experimental results show that BCAT and BCAT+ achieve better global adversarial robustness generalization performance than the state-of-the-art adversarial defense methods.

## 2. Related Works

### 2.1. Adversarial Training

AT is widely recognized as the only method that can improve the adversarial robustness of DNNs. Standard AT [4,5,9] is formulated as a min-max optimization problem. The inner maximization generates the worst-case adversarial examples using a first-order adversary called Projected Gradient Descent (PGD), and the outer minimization trains the model on the generated adversarial examples to update the model parameters. Many recently proposed state-of-the-art methods are based on this AT formulation. For example, Adversarial Logit pairing (ALP) [32] encourages the logits of a clean image x and its corresponding adversarial example $x^{\prime }$ to be similar. ALP imposes regularization on the model, which encourages similar feature distribution of clean and adversarial examples. TRADES [33] trains a model by optimizing a loss function consisting of two terms: one for maximizing the natural accuracy of the model, and another for improving the adversarial robustness of the model. TRADES provides a better trade-off between robustness and accuracy. TLA [34] and AT2L [35] combine metric learning with AT to train a model on triplet loss, which produces more robust classifiers. Zhang et al. [36] propose a feature scattering-based AT approach that considers inter-sample relationships for improving the adversarial robustness of DNNs. Yu et al. [37] demonstrate that latent features in an adversarially trained model are susceptible to adversarial attacks and propose the LAFEAT method to improve the robustness of the latent features against adversarial attacks. Chen et al. [38] propose self-supervised AT that maximizes the mutual information between the representations of clean examples and corresponding adversarial examples during training. Liu et al. [39] propose a defense algorithm named Adv-BNN that combines AT and the Bayesian neural network. Wang et al. [40] propose a dynamic training strategy to gradually increase the convergence quality of the generated adversarial examples, which improves the robustness of AT. Rice et al. [41] demonstrate that the improvement in adversarial robustness of AT can be achieved by simply adopting early stopping. Yu et al. [42] propose an AT-based method that can learn a representation that captures the shared information between clean examples and their corresponding adversarial examples while discarding these samples’ view-specific information, which leads to an improved robust vs. natural accuracy tradeoff.

Apart from these works that aim to improve the robustness generalization performance of AT, there are also many studies trying to solve specific problems in AT. For example, AT based on the min-max formulation hurts the standard generalization of DNNs. Zhang et al. [43] propose a novel formulation of AT called friendly adversarial training (FAT) that trains a model on the least adversarial examples instead of the worst-case adversarial examples. FAT achieves adversarial robustness without compromising natural generalization. It is known that adversarial robustness requires a larger capacity of a network than that for standard generalization [4,5,9]. In order to achieve compactness of the robust models, ADMM [44] and HYDRA [45] combine AT with weight pruning to give consideration to adversarial robustness and model compactness at the same time. Adversarial robustness gained from AT comes with a high computational cost. In order to reduce the computational cost of AT, freeAT [46] generates adversarial examples and updates model parameters within one gradient computation, thus speeding up the AT. YOPO [47] restricts most of the forward and backward propagation of AT within the first layer of a network during adversary updates, which reduces the computational cost. The single-step adversary such as FGSM can reduce the computational cost but fails to defend against adversarial attacks. Wong et al. [48] propose to train a model using FGSM combined with random initialization. Vivek et al. [49] propose a single-step AT method with dropout scheduling. Most of the works on adversarial robustness design methods are on balanced datasets. Wu et al. [50] investigate the adversarial vulnerability and defense under long-tailed distribution and propose RoBal which tackles adversarial robustness under long-tailed distribution.

### 2.2. Regularization

Regularization is any method we adopt to improve the generalization performance of a learning algorithm. There are many regularization methods. For example, $L_{2}$ regularization [26], also known as weight decay, adds a regularization term measuring the overall size of weight parameters by $L_{2}$ norm to the loss function to penalize high weight values, which prevents the model from getting too complex, thus reducing overfitting and improving generalization. Similar to $L_{2}$ regularization, $L_{1}$ regularization [51] replaces the $L_{2}$ norm with the $L_{1}$ norm to penalize the size of weight parameters, which results in a more sparse weight distribution. Other than the weight parameters, the penalty can also be applied to the activations of the units in DNNs to result in representational sparsity [52], which improves the generalization performance of DNNs. The lack of labeled data is a reason for the poor generalization performance of DNNs. Data augmentation adds synthetic data into the training set to improve sample complexity. DNNs trained on the augmented training set benefit from the improved sample complexity, thus the generalization performance is improved. The synthetic data can be acquired by applying geometric transformations such as flipping, cropping, rotation, and translation to already existing data [28] or be generated using Generative Adversarial Networks (GANs) [29]. When training models with a large capacity, the generalization error often peaks before the training is finished. Early stopping adopts the model parameters with the best generalization performance rather than the model parameters after the training is finished, which is a simple but effective regularization method. Caruana et al. [53] explain the regularization of early stopping imposed on restricting the model complexity: models with larger capacity first learn hypotheses that are similar to those learned by smaller models during the training process. When early stopping is used, the training of the larger model can be halted when the large model’s parameters are similar to parameters learned by smaller nets. Dropout [27] randomly masks out the hidden units of a network by multiplying their outputs by zero during training. This is similar to training an ensemble of different networks and then averaging the predictions of all networks, which improves the generalization performance of the single network. Other regularization methods such as semi-supervised learning [54], multitask learning [55], and noise injection [56] can also improve the generalization performance of DNNs.

## 3. Methods

### 3.1. Label-Feature Distribution Mismatch

**Figure 1:**
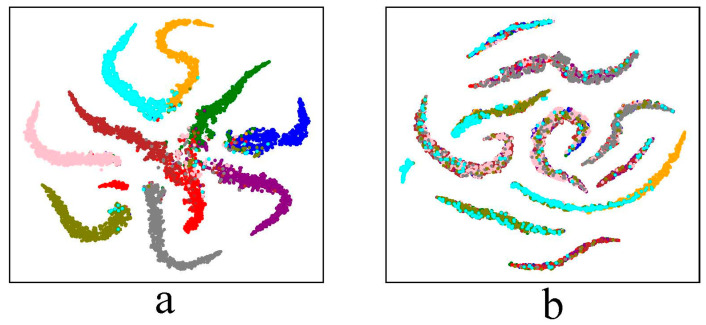
t-SNE visualization of the feature distribution of clean examples and adversarial examples of a standard-trained 11-layer CNN. Examples of the same color have the same ground-truth label. (**a**) Clean examples. (**b**) Adversarial examples.

An intriguing property of adversarial examples which we call the Label-Feature Distribution Mismatch is first introduced here. As can be seen from Figure 1, for a standard-trained 11-layer CNN model, the feature distribution of clean examples matches the ground-truth label distribution of clean examples. Namely, the features of clean examples that have the same ground-truth label exhibit similar spatial distribution in the feature space. However, when it comes to adversarial examples, the feature exhibits different spatial distribution from the ground-truth label distribution: the features of adversarial examples that have the same ground-truth label distributed in different areas in the feature space; and the features of adversarial examples that have different ground-truth labels may exhibit similar spatial distribution in the feature space. Due to the Label-Feature Distribution Mismatch property, adversarial examples can mislead the classification model to output incorrect classes because the perturbations move the adversarial examples across the classification boundary. Suppose $\left(x,\;y\right)\sim D$ be clean examples and corresponding ground-truth labels sampled from underlying data distribution D; $f:x\to \hat{y}$ is a classification model that outputs the predicted label $\hat{y}$ for the input x; $x^{\prime }$ is the adversarial example corresponding to x. In this paper, the ground-truth labels Y of the adversarial examples $X^{\prime }$ is called real labels, and the predicted labels $Y^{\prime }=f\left(X^{\prime }\right)$ of the adversarial examples $X^{\prime }$ is called fake labels.

### 3.2. Motivation

**Figure 2:**
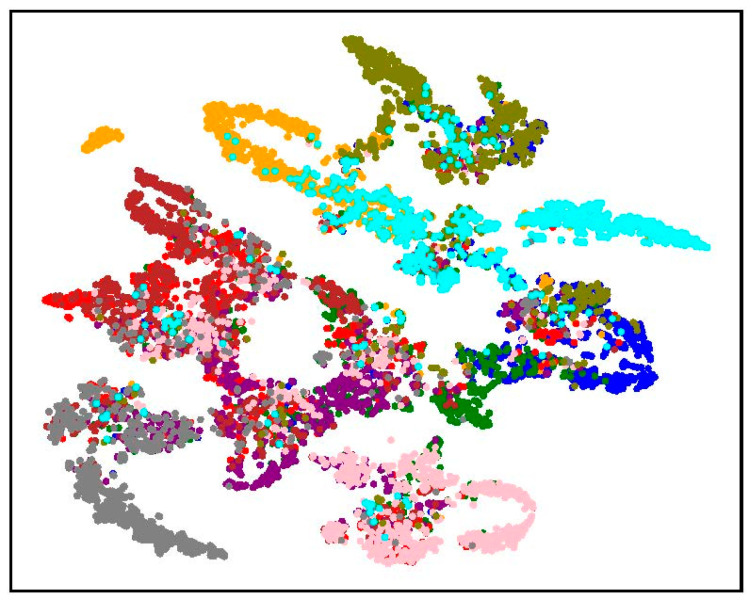
t-SNE visualization of the feature distribution of the adversarial examples of an 11-layer CNN trained by AT. Examples of the same color have the same ground-truth label.

AT [9] trains DNNs using online-generated worst-case adversarial examples. This training strategy imposes regularization on the distribution of adversarial examples to decrease the intra-class distance and increase the inter-class distance of the features of adversarial examples, as shown in Figure 2. As a result, AT mitigates the Label-Feature Distribution Mismatch problem of adversarial examples and improves the adversarial robustness generalization of DNNs. Therefore, the adversarial robustness generalization performance of DNNs can be improved from the point of view of regularizing the feature distribution. Nevertheless, the feature distribution of adversarial examples with the same real labels is still not ideal compared to clean examples (for example, the cyan points in Figure 2). BC-learning [31] is able to impose constraints on the feature distribution of clean examples to enlarge Fisher’s criterion and regularize the positional relationship among feature distributions, thus improving the standard generalization. Therefore, it is reasonable to assume that if BC-learning is applied to AT, the feature distribution of adversarial examples can be further regularized, and then the adversarial robustness generalization can be further improved.

### 3.3. BCAT: Between-Class Adversarial Training

In this section, we first introduce the standard AT formulation. Then, we propose the BCAT method and introduce how to apply BC-learning to AT.

Madry et al. [9] formulate AT as a min-max optimization problem:

(1) $$\underset{\theta }{\min}E_{\left(x,\;y\right)\sim D}\left[\underset{\|x^{\prime }-x\|\in S}{\max}L\left(\theta ,\;x^{\prime },\;y\right)\right]$$

where $S\subseteq {\mathbb{R}}^{d}$ is the set of perturbations the threat model can find, such as the union of the $L_{\infty }$-balls around the clean examples X. $L\left(\cdot \right)$ is a loss function such as the cross-entropy loss for DNNs. In this min-max optimization, the inner maximization problem finds adversarial examples that maximize the loss function, which is solved by PGD [9]:

(2) $$x^{t+1}=\prod_{x+S}\left(x^{t}+\alpha \mathrm{sgn}\left(\nabla_{x}L\left(\theta ,\;x,\;y\right)\right)\right)$$

**Figure 3:**
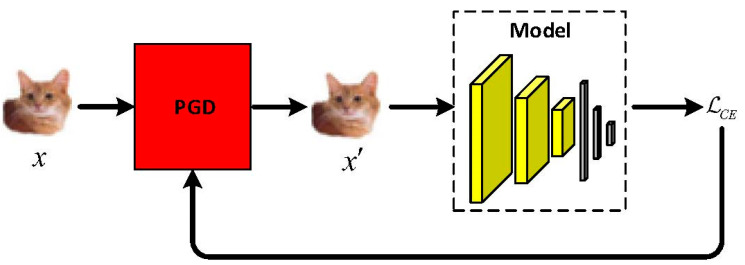
The training procedure of AT. In each training step: (1) the threat model attacks the model using PGD to generate adversarial examples; (2) the generated adversarial examples are used to train the model. This procedure is iterated until the model converges.

The outer minimization problem finds model parameters so that the loss function is minimized on the adversarial examples found by the inner maximization, which can be solved by back-propagation for DNNs. The training procedure of AT is exhibited in Figure 3.

As shown by Figure 3, there are two substeps in each training loop. First, the threat model attacks the model using PGD to generate adversarial examples $x^{\prime }$ from clean examples x according to the inner maximization of Equation (1). Second, the generated adversarial examples $x^{\prime }$ are used to train the model according to the outer minimization of Equation (1). This procedure is iterated until the model converges.

In order to regularize the feature distribution of adversarial examples and improve the adversarial robustness generalization performance of DNNs trained by AT, we propose the BCAT method which applies BC-learning to AT. First, adversarial examples $x^{\prime }$ are generated by the inner maximization of Equation (1):

(3) $$x^{\prime *}=\arg\underset{\|x^{\prime }-x\|\in S}{\max}L\left(\theta ,\;x^{\prime },\;y\right)$$

Suppose $x_{1}^{\prime }$ and $x_{2}^{\prime }$ are two adversarial examples with different real labels generated from Equation (3), and $y_{1}$ and $y_{2}$ are their one-hot real labels. Then, a random mixing ratio r is generated from $U\left(0,\;1\right)$, and two sets of adversarial examples and real labels are mixed with this mixing ratio:

(4) $$x_{mixed}^{\prime }=rx_{1}^{\prime }+\left(1-r\right)x_{2}^{\prime }$$

(5) $$y_{mixed}=ry_{1}+\left(1-r\right)y_{2}$$

Different from standard AT which trains the model on adversarial examples and their real labels, BCAT trains the model on mixed adversarial examples and mixed real labels. Namely, BCAT trains the model to output the mixing ratio of the mixed adversarial examples from different classes. This is by finding the trainable parameters θ of the DNN model that minimize the loss function L on the mixed adversarial examples $x_{mixed}^{\prime }$, and true labels $y_{mixed}$:

(6) $$\theta^{*}=\arg\underset{\theta }{\min}E_{\left(x_{mixed}^{\prime },\;y_{mixed}\right)\sim D_{mixed}}L\left(\theta ,\;x_{mixed}^{\prime },\;y_{mixed}\right)$$

According to [31], Kullback-Leibler divergence is adopted as the loss function for BCAT:

(7) $$L\left(\theta ,x_{mixed}^{\prime },y_{mixed}\right)=L_{KLD}\left(y_{mixed}∥y^{\prime };\theta \right)=\overset{N}{\underset{n=1}{\sum }}\overset{K}{\underset{k=1}{\sum }}y_{mixed}^{nk}\log\left(\frac{y_{mixed}^{nk}}{y^{\prime nk}}\right)$$

where $y^{\prime }$ is the output of the DNN model given the mixed adversarial examples $x_{mixed}^{\prime }$. Algorithm 1 describes the training procedure of BCAT.

**Algorithm 1** Pseudo code of BCATInput:Dataset D, initial weight parameters $\theta_{0}$, training steps K, batch size M, PGD perturbation value $\epsilon_{t}$, PGD step size α, PGD number of steps T
Output:

weight parameters θ

1For i=1, 2, …, K  do2 $\mathrm{Sample}\;\mathrm{two}\;\mathrm{batches}\;\left(x_{m}^{1},\;y_{m}^{1}\right)$$,\;\left(x_{m}^{2},\;y_{m}^{2}\right)$ with corresponding examples having different class label3 For j=1, 2, …, T  do4  For m=1, 2, …, M  do5   $x_{m}^{\prime 1}\leftarrow \arg\underset{\|x_{m}^{\prime 1}-x_{m}^{1}\|\in S}{\max}L\left(\theta ,\;x_{m}^{\prime 1},\;y_{m}^{1}\right)$
6   $x_{m}^{\prime 2}\leftarrow \arg\underset{\|x_{m}^{\prime 2}-x_{m}^{2}\|\in S}{\max}L\left(\theta ,\;x_{m}^{\prime 2},\;y_{m}^{2}\right)$
7  End for8 End for9 $\mathrm{Generate}\;a\;\mathrm{batch}\;\mathrm{of}\;\mathrm{random}\;\mathrm{mixing}\;\mathrm{ratio}\;r_{m}\sim U{\left(0,\;1\right)}^{m}$
10 For m=1, 2, …, M  do11  $x_{m}^{\prime \;mixed}=r_{m}x_{m}^{\prime \;1}+\left(1-r_{m}\right)x_{m}^{\prime \;2}$
12  $y_{m}^{mixed}=r_{m}y_{m}^{1}+\left(1-r_{m}\right)y_{m}^{2}$
13 End for14 $\theta_{i+1}\leftarrow \arg\underset{\theta_{i}}{\min}E_{\left(x^{\prime \;mixed},\;\;y^{mixed}\right)\sim D_{m}}L\left(\theta_{i},\;x^{\prime \;mixed},\;\;y^{mixed}\right)$
15End for

### 3.4. BCAT+: A More Powerful Mixing Method

Inspired by the mixing method of BC+ [31], we adopt another mixing method that treats images as waveform data. This mixing method is a modified version of the mixing method of BC+, which aims to better adapt to AT. In AT-based methods, pixel values of input data are normalized to a fixed range such as $\left[-1,\;1\right]$ because of bounded adversarial perturbations. However, in the mixing method for BC+, per-image mean values are first subtracted from images, and then the zero-centered images are normalized for each channel using the mean and standard deviation calculated from the whole training data. In order to better adapt to AT, we do not adopt the normalization method of BC+ for BCAT+, and simply restrict the pixel values of images to $\left[-1,\;1\right]$. Specifically, two normalized adversarial examples $x_{1}^{\prime }$ and $x_{2}^{\prime }$ are first mixed by Equation (8) instead of Equation (4):

(8) $$x_{mixed}^{\prime }=\frac{rx_{1}^{\prime }+\left(1-r\right)x_{2}^{\prime }}{\sqrt{r^{2}+{\left(1-r\right)}^{2}}}$$

Equation (8) takes waveform energy into consideration, which is proportional to the square of the amplitude. This mixing method prevents the input variance from decreasing. Second, following [31], we consider the difference of energies of two adversarial examples and use a new coefficient p instead of r to mix two adversarial examples by

(9) $$x_{mixed}^{\prime }=\frac{px_{1}^{\prime }+\left(1-p\right)x_{2}^{\prime }}{\sqrt{p^{2}+{\left(1-p\right)}^{2}}}$$

where p is solved from $p\sigma_{1}:\left(1-p\right)\sigma_{2}=r:\left(1-r\right)$:

(10) $$p=\frac{1}{1+\frac{\sigma_{1}}{\sigma_{2}}\cdot \frac{1-r}{r}}$$

where $\sigma_{1}$ and $\sigma_{2}$ are the standard deviations per image.

The main differences between BCAT+ and BC+ are that BCAT+ uses adversarial examples instead of clean examples and restricts the pixel values of adversarial examples into range $\left[-1,\;1\right]$. The advantage of BCAT+ over BCAT can be explained from two aspects. Firstly, BCAT simply mixes two adversarial examples by linear combination, but BCAT+ mixes two adversarial examples by treating them as waveform data. CNNs have an aspect of treating input data as waveforms. Therefore, the mixed adversarial examples from BCAT+ are more adaptive to CNNs than those from BCAT; Secondly, the mixing methods of both BCAT and BCAT+ are, by nature, two data augmentation methods which increase the variance of the training data, which imposes constraints on the feature distribution of the adversarial examples, and thus improves the adversarial robustness generalization performance. The key point is that the mixing method of BCAT+ takes the difference in the energies of the adversarial examples into consideration, which can generate mixed adversarial examples with higher variance. This is equivalent to imposing stronger constraints on the feature distribution of the adversarial examples. Experiments in Section 4 will demonstrate the advantage of BCAT+ over BCAT in terms of adversarial robustness generalization performance. Algorithm 2 describes the training procedure of BCAT+.

**Algorithm 2** Pseudo code of BCAT+Input:Dataset D, initial weight parameters $\theta_{0}$, training steps K, batch size M, PGD perturbation value $\epsilon_{t}$, PGD step size α, PGD number of steps T
Output:weight parameters θ
1For i=1, 2, …, K  do2 $\mathrm{Sample}\;\mathrm{two}\;\mathrm{batches}\;\left(x_{m}^{1},\;y_{m}^{1}\right)$$,\;\left(x_{m}^{2},\;y_{m}^{2}\right)$ with corresponding examples having different class labels3 For j=1, 2, …, T  do4  For m=1, 2, …, M do5   $x_{m}^{\prime 1}\leftarrow \arg\underset{\|x_{m}^{\prime 1}-x_{m}^{1}\|\in S}{\max}L\left(\theta ,\;x_{m}^{\prime 1},\;y_{m}^{1}\right)$
6   $x_{m}^{\prime 2}\leftarrow \arg\underset{\|x_{m}^{\prime 2}-x_{m}^{2}\|\in S}{\max}L\left(\theta ,\;x_{m}^{\prime 2},\;y_{m}^{2}\right)$
7  End for8 End for9 $\mathrm{Generate}\;a\;\mathrm{batch}\;\mathrm{of}\;\mathrm{random}\;\mathrm{mixing}\;\mathrm{ratio}\;r_{m}\sim U{\left(0,\;1\right)}^{m}$
10 $p_{m}\leftarrow \frac{1}{1+\frac{\sigma_{m}^{1}}{\sigma_{m}^{2}}\cdot \frac{1-r_{m}}{r_{m}}}$
11 For m=1, 2, …, M do12  $x_{m}^{\prime \;mixed}\leftarrow \frac{p_{m}x_{m}^{\prime 1}+\left(1-p_{m}\right)x_{m}^{\prime 2}}{\sqrt{p_{m}^{2}+{\left(1-p_{m}\right)}^{2}}}$
13  $y_{m}^{mixed}=r_{m}y_{m}^{1}+\left(1-r_{m}\right)y_{m}^{2}$
14 End for15 $\theta_{i+1}\leftarrow \arg\underset{\theta_{i}}{\min}E_{\left(x^{\prime \;mixed},\;\;y^{mixed}\right)\sim D_{m}}L\left(\theta_{i},\;x^{\prime \;mixed},\;\;y^{mixed}\right)$
16End for

As two training algorithms for DNNs, BCAT and BCAT+ iteratively update the parameters θ of the DNN models to minimize the loss function L by stochastic gradient descent. In each training iteration, firstly the gradients of the loss function L with respect to the parameters θ are calculated; then, the parameters θ are updated for one small step along the opposite direction of the gradients, which is the direction in which the value of the loss function descends the fastest. There is the parameters updating rule:

(11) $$\theta^{i+1}=\theta^{i}-\eta \frac{\partial L}{\partial \theta^{i}}$$

where $\theta^{i}$ are the parameters calculated in the ith iteration during training; η is the learning rate that controls how much to change the model in each training iteration. η is a critical hyperparameter that affects the convergence of the training process, as too large η may result in a sub-optimal set of parameters too fast or cause the training not to converge, whereas too small η may result in a long training process that could become stuck. The chosen value of η is introduced in Section 4.3, and the convergence process of BCAT and BCAT+ is visualized and analyzed in Section 4.4.3.

**Figure 4:**
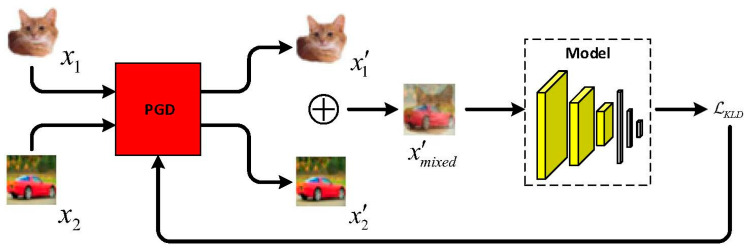
The training procedure of BCAT (+). In each training step, (1) the threat model attacks the model using PGD to generate adversarial examples; (2) the adversarial examples having different true labels are mixed two by two; (3) the mixed adversarial examples are used to train the model to output the mixing ratio. This procedure is iterated until the model converges.

**Figure 5:**
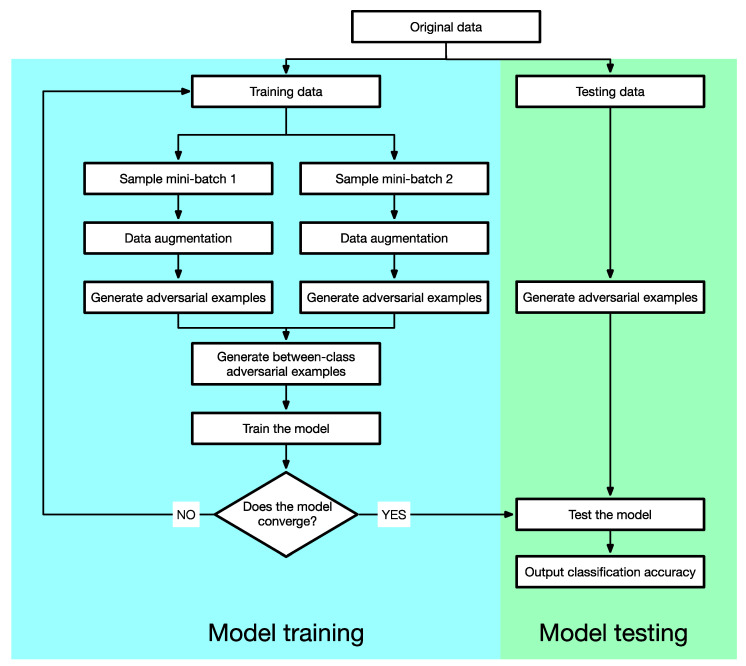
Overall framework of the proposed method.

The training procedure of BCAT (+) is exhibited in Figure 4. There are three substeps in each training step. First, the threat model attacks the model using PGD to generate adversarial examples $x^{\prime }$ from clean examples x according to the inner maximization of Equation (1). Second, the adversarial examples having different true labels are mixed two by two to generate mixed adversarial examples $x_{mixed}^{\prime }$. Third, the mixed adversarial examples $x_{mixed}^{\prime }$ are used to train the model to output the mixing ratio. This procedure is iterated until the model converges. The overall framework representing the working mechanism of the proposed method is shown in Figure 5. The classification model is first trained using BCAT (+) on the training data. The adversarial robustness generalization performance of the trained model is then evaluated on the unseen testing data.

### 3.5. Real Label and Fake Label

**Figure 6:**
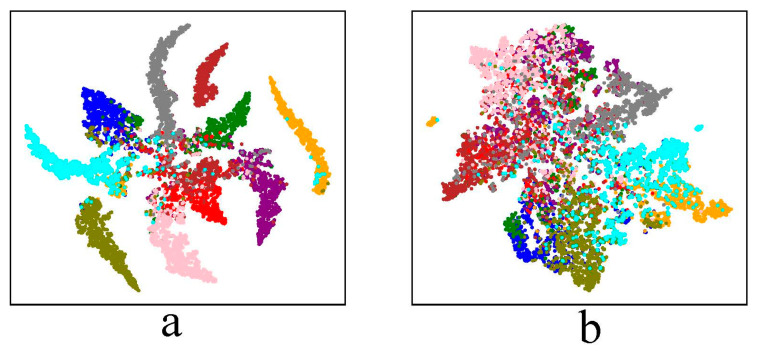
t-SNE visualization of the feature distribution of clean examples and adversarial examples of an 11-layer CNN trained for 100 epochs. Examples of the same color have the same ground-truth label. (**a**) Clean examples of the standard trained model. (**b**) Adversarial examples of the adversarially trained model.

Recall that when BC-learning is applied to clean examples, two clean examples from different classes are chosen to be mixed by a random ratio. This operation chooses examples from different spatial distributions and regularizes the feature distribution of clean examples by training the model on the mixed clean examples and labels. However, when it comes to adversarial examples, circumstances change due to the Label-Feature Distribution Mismatch problem we introduce in Section 3.1 because adversarial examples with different real labels may have similar spatial distributions and adversarial examples with the same real labels may have different distributions in the feature space. Even when the AT is finished, the Label-Feature Distribution Mismatch problem still exists, according to Figure 2; yet, the feature distribution of clean examples of a model trained for 100 epochs already matches with the ground-truth label distribution, according to Figure 6. Therefore, we also consider a different realization of BCAT and BCAT+ that takes the fake label of adversarial examples into consideration. Specifically, the two adversarial examples chosen to be mixed have different real labels and fake labels. We call BCAT and BCAT+ methods taking fake labels into consideration as BCATf and BCATf+, respectively. Due to the higher computational complexity of BCATf and BCATf+ than BCAT and BCAT+, and considering BCAT and BCAT+ have already achieved good adversarial robustness generalization performance, we mainly focus on BCAT and BCAT+ in our experiments and show the performance of BCATf and BCATf+ in Section 4.6.1.

## 4. Results and Discussion

### 4.1. Datasets

We evaluated BCAT and BCAT+ on CIFAR-10, CIFAR-100, and SVHN datasets in this paper. The CIFAR-10 dataset contains 60,000 32 × 32 RGB images from 10 classes: airplane, automobile, bird, cat, deer, dog, frog, horse, ship, and truck. The training set contains 50,000 samples and the testing set contains 10,000 samples. The CIFAR-100 dataset has 60,000 32 × 32 RGB images from 100 classes. There are 500 training images and 100 testing images per class. The 100 classes in the CIFAR-100 are grouped into 20 superclasses. Each image comes with a “fine” label (the class to which it belongs) and a “coarse” label (the superclass to which it belongs). The SVHN dataset contains 10 classes of street view house numbers RGB images of size 32 × 32. The training set contains 73,257 samples and the testing set contains 26,032 samples. Pixel values are normalized to $\left[-1,\;1\right]$ for these three datasets in this paper. During training, the standard data augmentation scheme [57] is applied to CIFAR-10 and CIFAR-100 datasets.

### 4.2. Threat Model

The threat model used in this paper for evaluating the adversarial robustness generalization of the proposed method is the $L_{\infty }$-PGD, which generates $L_{\infty }$ norm bounded adversarial examples against the defended networks. The number of steps and the step size of PGD are set to be 20 and 2/255. A wide range of perturbation values is chosen from 1/255 to 8/255 with a step size of 1/255 to globally evaluate the adversarial robustness generalization. Both white-box attacks and black-box attacks are considered in our experiments.

### 4.3. Training Parameters

The same training schedule is adopted for CIFAR-10, CIFAR-100, and SVHN in this paper. The batch size is set to 128 and the epoch is set to 250. The Momentum optimizer parameterized with a momentum of 0.9 is used and the Nesterov is used for the optimizer. The initial learning rate is set to be 0.1 and is decayed by a factor of 10 at epochs $\left\{100,\;150,\;200\right\}$. Weight decay is set to 0.0005 according to [58], which is shown to achieve higher adversarial robustness generalization accuracy.

### 4.4. Evaluation under White-Box Attacks

In this section, we evaluate BCAT and BCAT+ under white-box attacks and compare BCAT and BCAT+ with several strong baselines (standard AT [9], freeAT [46], ALP [32], TRADES [33], and Yu et al.’s method [42]) on CIFAR-10, CIFAR-100, and SVHN to illustrate the superiority of BCAT and BCAT+ in terms of improving the adversarial robustness generalization of DNNs. For convenience, we denote Yu et al.’s method as ATLSI (Adversarial Training by Learning Shared Information) in this paper.

#### 4.4.1. Feature Distribution

**Figure 7:**
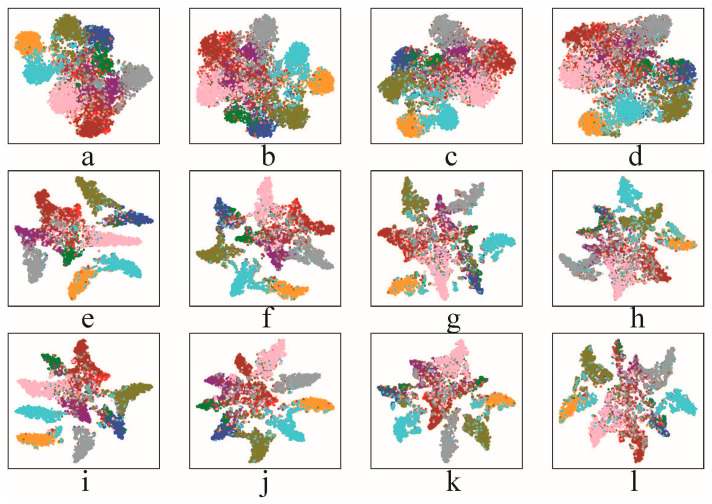
t-SNE visualization of the feature distribution of adversarial examples of ResNet34 networks trained using (**a**–**d**) standard AT, (**e**–**h**) BCAT, and (**i**–**l**) BCAT+ on CIFAR-10.

As introduced in Section 3.2, BC-learning can benefit the standard generalization of DNNs by regularizing the feature distribution of clean examples to yield larger inter-class distances and smaller inner-class distances. To study whether BC-learning can also regularize the feature distribution of adversarial examples after combining with AT, we first train three ResNet34 networks on CIFAR-10 using standard AT, BCAT, and BCAT+, respectively, and then use t-SNE to visualize the testing set adversarial examples of CIFAR-10 generated on these three ResNet34 networks in a two-dimensional feature space. The visualization results are displayed in Figure 7. As can be seen from the top row of Figure 7, for the network trained using standard AT, the feature distributions of the adversarial examples from different classes significantly overlap in feature space; there still exists a noticeable label-feature distribution mismatch problem in the network trained using standard AT. By contrast, as shown in the middle row and the bottom row of Figure 7, the networks trained using BCAT and BCAT+ exhibit better feature distribution than standard AT: the inter-class distance is substantially increased, and the inner-class distance is substantially decreased. The label-feature distribution mismatch problem is effectively mitigated by BCAT and BCAT+. This indicates that BC-learning imposes effective regularization on the feature distribution of the adversarial examples. In fact, a larger discrimination margin allows the networks to learn better classification boundaries during AT, thus improving the adversarial robustness generalization.

#### 4.4.2. Robustness Generalization

**Figure 8:**
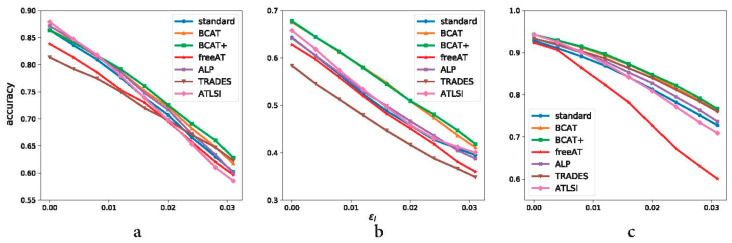
Robustness generalization curves of different defense methods on CIFAR-10, CIFAR-100, and SVHN. ResNet34, WRN34–5, and ResNet18 are used for CIFAR-10, CIFAR-100, and SVHN, respectively. (**a**) CIFAR-10. (**b**) CIFAR-100. (**c**) SVHN.

In order to quantitatively analyze the robustness generalization and the standard generalization performance of BCAT and BCAT+ and compare them with the baselines, we first train several networks using BCAT, BCAT+, and the baselines on CIFAR-10, CIFAR-100, and SVHN. Specifically, ResNet34 and ResNet18 are adopted for CIFAR-10 and SVHN. WRN34–5 is adopted for CIFAR-100. The perturbation value of all evaluated defense methods during training is set to be 8/255 for CIFAR and 12/255 for SVHN. The number of steps and step size are set to be 7 and 2/255. Then, we test the robustness generalization accuracy and the standard generalization accuracy of these networks under PGD20 with a wide range of perturbation values described in Section 4.2. The experimental results are exhibited in Table 1, Table 2 and Table 3 and Figure 8.

**CIFAR-10** Results on CIFAR-10 are exhibited in Table 1 and Figure 8a. For the sake of simplicity, the results under only four representative perturbation values of 2/255, 4/255, 6/255, and 8/255 are listed in Table 1. As shown in Table 1, for the ResNet34 network, BCAT+ achieves the highest robustness generalization accuracy among the evaluated defense methods at all chosen perturbation values except for the perturbation value of 2/255, where ATLSI slightly outperforms BCAT+ (0.818 vs. 0.817). The robustness generalization accuracy of BCAT ranks second at the perturbation values of 4/255 and 6/255; at the local perturbation values of 2/255 and 8/255, ATLSI, ALP, and TRADES slightly outperform BCAT, respectively. In a word, for the ResNet34 network, BCAT and BCAT+ outperform the baselines in terms of global robustness generalization performance, which is considered a more convincing evaluation metric than the robustness generalization performance at a single perturbation value [59]. For the ResNet18 network that has a smaller capacity, the robustness generalization accuracy of BCAT+ ranks first only at the perturbation values of 4/255, 6/255, and 8/255; at the small perturbation value of 2/255, ATLSI achieves the highest robustness generalization accuracy. The robustness generalization accuracy of BCAT ranks second only at the perturbation value of 8/255. Comparing the ResNet34 network and the ResNet18 network indicates that a larger network capacity can benefit the robustness generalization performance of BCAT and BCAT+. In terms of standard generalization, nevertheless, the performance of BCAT and BCAT+ is less than ideal on CIFAR-10; ATLSI achieves the highest standard generalization accuracy on the ResNet34 network and the ResNet18 network. A reasonable explanation is that this is due to the specificity of the datasets. Figure 8a shows the superiority of BCAT+ in robustness generalization.

**CIFAR-100** Results on CIFAR-100 are shown in Table 2 and Figure 8b. As listed in Table 2, BCAT+ and BCAT achieve the highest and the second-highest standard generalization accuracy among the evaluated defense methods; recalling the specificity of datasets mentioned above, the standard generalization performance gain of BCAT and BCAT+ on CIFAR-100 is more noticeable (3.1% and 3.4%) than that on CIFAR-10. BCAT and BCAT+ also achieve significantly higher robustness generalization accuracy than the baseline methods for all the chosen perturbation values. Figure 8b intuitively shows the superiority of BCAT+ and BCAT. Compared to CIFAR-10, the performance gap between BCAT (+) and the baseline methods are larger on CIFAR-100.

**SVHN** Results on SVHN are shown in Table 3 and Figure 8c. As can be seen from Table 3, for both the ResNet34 network and the ResNet18 network, BCAT+ and BCAT achieve the highest and the second-highest standard generalization accuracy and robustness generalization accuracy among the evaluated defense methods under the majority of chosen perturbation values, except for the perturbation value of 8/255 for the ResNet34 network where ATLSI outperforms BCAT+. Nonetheless, the global robustness generalization performance of BCAT and BCAT+ is not affected by this exception. Comparing the robustness generalization accuracy for the ResNet34 network and the ResNet18 network, we find that the ResNet18 network outperforms the ResNet34 network for AT, freeAT, and ALP; for TRADES, BCAT, and BCAT+, the ResNet18 network outperforms the ResNet34 network for higher perturbation values. This observation is different from that of CIFAR-10 where the network with a larger capacity performs better than the network with a smaller capacity. This is because classification on SVHN is easier than classification on CIFAR-10. A bigger network is easy to overfit on SVHN. Figure 8c exhibits the standard and robustness generalization accuracy for ResNet18, from which we can see the superiority of BCAT+.

#### 4.4.3. Convergence Analysis

**Figure 9:**
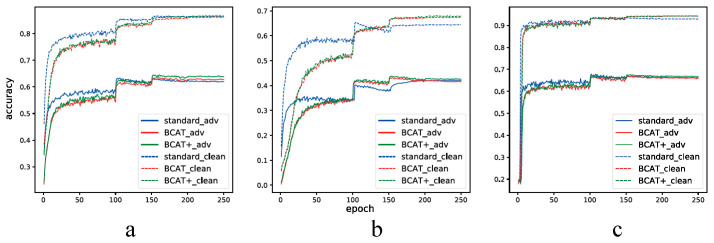
The convergence of the testing accuracy on clean examples and adversarial examples for CIFAR-10, CIFAR-100, and SVHN. ResNet34, WRN34–5, and ResNet18 are used for CIFAR-10, CIFAR-100, and SVHN, respectively. (**a**) CIFAR-10. (**b**) CIFAR-100. (**c**) SVHN.

We also analyze the convergence process of the robustness and standard generalization accuracy of the networks trained using standard AT, BCAT, and BCAT+ on CIFAR-10, CIFAR-100, and SVHN datasets. The networks used for CIFAR-10, CIFAR-100, and SVHN are ResNet34, WideNetNet34–5, and ResNet18, respectively. During training, the step size of PGD is set to be 7 for the validation set. The validation accuracy during training is plotted in Figure 9.

**CIFAR-10** From Figure 9a it can be seen that, in the CIFAR-10 dataset, the robustness generalization accuracy of standard AT is higher than that of BCAT and BCAT+ before the first decay of the learning rate. After the first decay of the learning rate, the robustness generalization accuracy of BCAT+ gradually exceeds that of standard AT. After the second decay of the learning rate, the robustness generalization accuracy of BCAT exceeds that of standard AT. Additionally, the standard generalization of standard AT is exceeded by that of BCAT+ after the third decay of the learning rate.

**CIFAR-100** From Figure 9b it can be seen that, in the CIFAR-100 dataset, the robustness generalization accuracy of BCAT and BCAT+ exceed that of standard AT after the first decay of the learning rate. Although the robustness generalization accuracy of standard AT exceeds that of BCAT again after the third decay of the learning rate, BCAT+ ranks first until the end of the training process. Additionally, the standard generalization accuracy of BCAT and BCAT+ gradually exceeds that of standard AT after the first decay of the learning rate and keeps a large margin until the end of the training process.

**SVHN** As can be seen from Figure 9c, in the SVHN dataset, the robustness generalization accuracy of BCAT+ exceeds that of standard AT after the second decay of the learning rate. Additionally, the standard generalization accuracy of BCAT and BCAT+ exceeds that of standard AT after the second decay of the learning rate.

The observation above suggests that large training epochs and a proper learning rate schedule are vital to BCAT and BCAT+.

### 4.5. Evaluation under Black-Box Attacks

In order to demonstrate that the robustness of BCAT and BCAT+ is not a result of obfuscated gradients [23], we evaluate BCAT and BCAT+ under black-box attacks in this section. In black-box attacks, the adversary has access to nothing but the output of the target model; yet, due to the transferability of adversarial examples, the adversary can first construct a substitute model (or source model) and attack this substitute model to generate adversarial examples in the manner of white-box attacks. Then, the adversary attacks the black-box target model using the generated adversarial examples. According to [23], a robust model that does not rely on obfuscated gradients has better black-box robustness than white-box robustness. In our black-box attacks experiment, we adopt the defense-agnostic adversary; namely the substitute model constructed by the adversary is undefended. We first use PGD20 to generate adversarial examples with perturbation values of 2/255, 4/255, 6/255, and 8/255 on the substitute model, and then attack the defended target models with the generated adversarial examples. The perturbation value of all evaluated defense methods during training is set to be 8/255 for CIFAR and 12/255 for SVHN. The number of steps and step size are set to be 7 and 2/255. The experimental results on CIFAR-10, CIFAR-100, and SVHN are summarized in Table 4, Table 5, and Table 6.

**CIFAR-10** The data in Table 4 show that, on CIFAR-10, BCAT and BCAT+ achieve higher black-box robustness generalization accuracy than the white-box robustness generalization accuracy. Additionally, BCAT+ achieves the highest black-box robustness generalization accuracy among the evaluated defense methods under most of the perturbation values, with the exception of 4/255 where the standard AT slightly outperforms BCAT+.

**CIFAR-100** The data in Table 5 show that, on CIFAR-100, BCAT and BCAT+ achieve higher black-box robustness generalization accuracy than the white-box robustness generalization accuracy. BCAT+ and BCAT achieve the highest and the second-highest black-box robustness generalization accuracy under all chosen perturbation values.

**SVHN** From Table 6 it can be seen that, on SVHN, BCAT and BCAT+ achieve higher black-box robustness generalization accuracy than the white-box robustness generalization accuracy. Additionally, BCAT+ achieves the highest black-box robustness generalization accuracy under all chosen perturbation values. BCAT achieves the same black-box robustness generalization accuracy under perturbation values of 2/255, 4/255, and 6/255.

From the observation above, we know that our proposed BCAT+ outperforms the baselines in black-box attacks; this suggests that BCAT and BCAT+ do not rely on obfuscated gradients.

### 4.6. Ablation Study

In this section, we conduct ablation studies to investigate the effect of the fake label, data augmentation, and attack steps on the performance of BCAT and BCAT+.

#### 4.6.1. BCAT (+) and BCATf (+)

**Figure 10:**
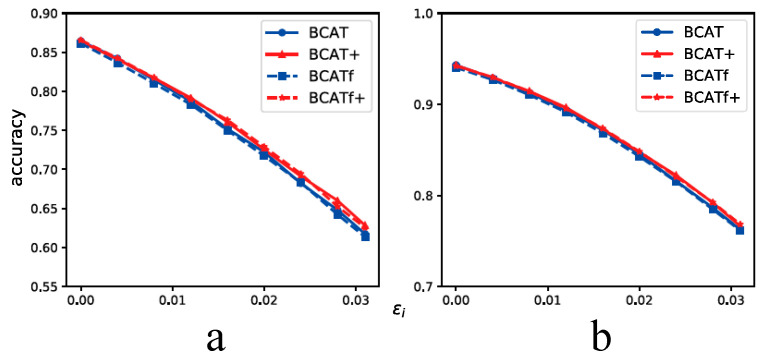
Standard generalization and robustness generalization curves of BCAT (+) and BCATf (+) on CIFAR-10 and SVHN. (**a**) CIFAR-10. (**b**) SVHN.

Previously, in Section 3.5, we introduce a different realization of BCAT and BCAT+ that we call BCATf and BCATf+. BCATf and BCATf+ take the fake label of adversarial examples into consideration. Specifically, two adversarial examples chosen to be mixed have different real labels and fake labels. Here, we conduct white-box attacks experiments to evaluate the standard generalization and the robustness generalization performance of BCATf and BCATf+ and compare BCATf and BCATf+ with BCAT and BCAT+ to study the effect of the fake label on BCATf and BCATf+. Because of the high computational cost of BCATf and BCATf+, we only conduct experiments on CIFAR-10 and SVHN. The perturbation value of BCAT (+) and BCATf (+) during training is set to be 8/255 for CIFAR-10 and 12/255 for SVHN. The number of steps and step size are set to be 7 and 2/255. The comparison results between BCAT (+) and BCATf (+) are given in Figure 10. From Figure 10, it can be seen that BCATf and BCATf+ achieve similar standard generalization and robustness generalization performance with BCAT and BCAT+ under different perturbation values. This observation implies that when applying BC-learning to AT, only considering real-label is enough for regularizing the feature distribution of adversarial examples. The fake label has no obvious effect on further improving the generalization performance.

#### 4.6.2. Ablation on Data Augmentation

As mentioned in the Introduction, BC-learning is an effective data augmentation method for AT, which adds synthetic mixed adversarial examples into original adversarial examples to improve the sample complexity. In order to study the relative importance between BC-learning and the standard data augmentation applied to CIFAR-10 and CIFAR-100, we compare the model using both methods and the model using only one of the two methods. The perturbation value during training is set to be 8/255. The number of steps and the step size are set to be 7 and 2/255. The experimental results are summarized in Table 7. In Table 7, ‘standard with/without’ stands for the adversarially trained models only using standard data augmentation or without any data augmentation. ‘BCAT (+) with/without’ stands for the adversarially trained models using both BC-learning and standard data augmentation or only using BC-learning. We can obtain two insights from the results. First, both BC-learning and standard data augmentation alone can improve the robustness and standard generalization performance of adversarially trained models on CIFAR-10 and CIFAR-100. However, the accuracy improvement gained from BC-learning is lower than that gained from standard data augmentation. Especially for high perturbation values such as 6/255 and 8/255, BC-learning alone even degrades the robustness generalization accuracy. Second, when BC-learning is used in conjunction with standard data augmentation, the resulting robustness generalization accuracy and the standard generalization accuracy are higher than that when using BC-learning or standard data augmentation alone. This indicates that standard data augmentation is vital when applying BC-learning to AT.

#### 4.6.3. Ablation on Attack Steps

**Figure 11:**
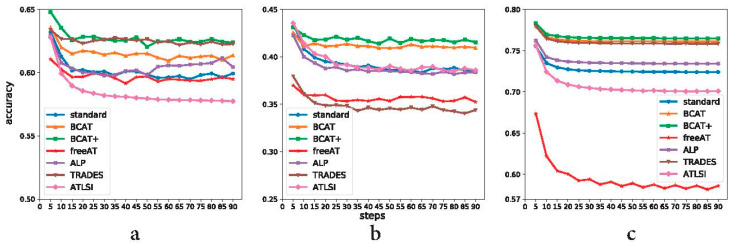
Robustness generalization curves of different defense methods under different attack steps on CIFAR-10, CIFAR-100, and SVHN. ResNet34, WRN34–5, and ResNet18 are used for CIFAR-10, CIFAR-100, and SVHN, respectively. (**a**) CIFAR-10. (**b**) CIFAR-100. (**c**) SVHN.

We also attack BCAT (+) and the baselines using a range of attack steps to show the effectiveness of our method under different attack strengths. The attack steps are chosen from 5 to 90 with a step size of 5. The attack step size is set to be 2/255. The perturbation value during training and testing is set to be 8/255. The results are shown in Figure 11. For CIFAR-10, except for attack steps 15, 35, 40, and 50 where TRADES slightly outperforms BCAT+, BCAT+ achieves better robustness generalization performance than the baselines under other attack steps. The overall performance of BCAT+ under the full spectrum of tested attack steps is better than TRADES. For CIFAR-100 and SVHN, BCAT+ significantly outperforms the baselines under the full spectrum of tested attack steps. Moreover, while the attack step is gradually increased, the robustness generalization accuracy of all the evaluated defense methods first decreases and then becomes stable. This also demonstrates that the evaluated defense methods are free of obfuscated gradients.

## 5. Conclusions

In this paper, we proposed two novel adversarial defense algorithms against both white-box and black-box attacks called BCAT and BCAT+ that combine BC-learning with standard AT. BCAT and BCAT+ first mix two adversarial examples that have different real labels using different mixing methods, and then train the DNN model on the mixed adversarial examples instead of on the original adversarial examples to output the mixing rate during AT, which mitigates the Label-Feature Distribution Mismatch problem of adversarial examples and improves the robustness generalization performance of the DNN model trained by AT. We evaluated BCAT and BCAT+ under white-box and black-box attacks on CIFAR-10, CIFAR100, and SVHN datasets. The experimental results show that BCAT and BCAT+ can effectively regularize the feature distribution of adversarial examples, thus achieving better global robustness generalization performance than the state-of-the-art adversarial defense methods.

The proposed methods still have some limitations. Firstly, the process of searching the adversarial examples of different real labels increases the computational cost of standard AT. Secondly, the step of mixing two adversarial examples is between the generation of adversarial examples and the update of weight parameters, which makes it difficult to reduce the computational cost of BCAT and BCAT+ by combining the generation of adversarial examples and the update of weight parameters. Therefore, in the future, we will design a method to reduce the computational cost of BCAT and BCAT+, while not significantly damaging the robustness generalization. Additionally, we will develop a more powerful mixing method to further improve the standard generalization and the robustness generalization performance of BCAT+.

## Figures and Tables

**Table 1:** Comparing the white-box adversarial robustness generalization accuracy of different defense methods on CIFAR-10.

Network	Defense	$\epsilon_{t}$	0	2/255	4/255	6/255	8/255
ResNet34	AT	8/255	0.863	0.809	0.739	0.666	0.602
	freeAT	8/255	0.838	0.786	0.730	0.658	0.597
	ALP	8/255	0.872	0.815	0.748	0.673	0.600
	TRADES	8/255	0.813	0.774	0.720	0.671	0.623
	ATLSI	8/255	**0.879**	**0.818**	0.739	0.654	0.586
	BCAT	8/255	0.863	0.814	0.752	0.682	0.617
	BCAT+	8/255	0.863	0.817	**0.761**	**0.691**	**0.628**
ResNet18	AT	8/255	0.850	0.799	0.739	0.673	0.604
	freeAT	8/255	0.823	0.770	0.712	0.644	0.582
	ALP	8/255	0.862	0.805	0.738	0.663	0.592
	TRADES	8/255	0.789	0.744	0.701	0.649	0.607
	ATLSI	8/255	**0.872**	**0.815**	0.739	0.650	0.570
	BCAT	8/255	0.841	0.794	0.735	0.671	0.610
	BCAT+	8/255	0.841	0.795	**0.741**	**0.678**	**0.611**

**Table 2:** Comparing the white-box adversarial robustness generalization accuracy of different defense methods on CIFAR-100.

Network	Defense	$\epsilon_{t}$	0	2/255	4/255	6/255	8/255
ResNet34-5	AT	8/255	0.644	0.565	0.488	0.427	0.395
	freeAT	8/255	0.628	0.559	0.482	0.418	0.360
	ALP	8/255	0.642	0.658	0.499	0.436	0.388
	TRADES	8/255	0.583	0.513	0.447	0.388	0.349
	ATLSI	8/255	0.659	0.574	0.496	0.430	0.400
	BCAT	8/255	0.675	0.612	**0.548**	0.474	0.411
	BCAT+	8/255	**0.678**	**0.614**	0.544	**0.480**	**0.418**

**Table 3:** Comparing the white-box adversarial robustness generalization accuracy of different defense methods on SVHN.

Network	Defense	$\epsilon_{t}$	0	2/255	4/255	6/255	8/255
ResNet34	AT	12/255	0.925	0.881	0.827	0.766	0.718
	freeAT	12/255	0.927	0.470	0.339	0.282	0.256
	ALP	12/255	0.929	0.891	0.838	0.775	0.720
	TRADES	12/255	0.938	0.907	0.864	0.813	0.758
	ATLSI	12/255	0.942	0.897	0.845	0.807	0.786
	BCAT	12/255	0.946	0.915	0.873	0.815	0.755
	BCAT+	12/255	**0.948**	**0.917**	**0.876**	**0.819**	**0.759**
ResNet18	AT	12/255	0.929	0.891	0.842	0.782	0.727
	freeAT	12/255	0.924	0.864	0.782	0.672	0.600
	ALP	12/255	0.934	0.900	0.853	0.795	0.737
	TRADES	12/255	0.933	0.904	0.863	0.812	0.760
	ATLSI	12/255	**0.943**	0.902	0.842	0.771	0.709
	BCAT	12/255	**0.943**	0.913	0.872	0.816	0.763
	BCAT+	12/255	**0.943**	**0.914**	**0.873**	**0.822**	**0.766**

**Table 4:** Comparing the black-box adversarial robustness generalization accuracy of different defense methods on CIFAR-10.

Source/Target Model	Defense	$\epsilon_{t}$	2/255	4/255	6/255	8/255
ResNet34/ResNet34	AT	8/255	0.862	**0.863**	0.856	0.855
	freeAT	8/255	0.836	0.835	0.832	0.828
	ALP	8/255	0.858	0.856	0.854	0.854
	TRADES	8/255	0.811	0.811	0.807	0.809
	BCAT	8/255	0.861	0.860	0.855	0.855
	BCAT+	8/255	**0.864**	0.861	**0.858**	**0.858**

**Table 5:** Comparing the black-box adversarial robustness generalization accuracy of different defense methods on CIFAR-100.

Source/Target Model	Defense	$\epsilon_{t}$	2/255	4/255	6/255	8/255
ResNet34-5/ResNet34-5	AT	8/255	0.640	0.636	0.636	0.634
	freeAT	8/255	0.622	0.619	0.618	0.617
	ALP	8/255	0.639	0.634	0.635	0.634
	TRADES	8/255	0.583	0.579	0.575	0.578
	BCAT	8/255	0.673	0.668	0.667	0.664
	BCAT+	8/255	**0.677**	**0.673**	**0.668**	**0.669**

**Table 6:** Comparing the black-box adversarial robustness generalization accuracy of different defense methods on SVHN.

Source/Target Model	Defense	$\epsilon_{t}$	2/255	4/255	6/255	8/255
ResNet18/ResNet18	AT	12/255	0.923	0.918	0.914	0.910
	freeAT	12/255	0.917	0.912	0.908	0.904
	ALP	12/255	0.929	0.923	0.918	0.914
	TRADES	12/255	0.928	0.923	0.918	0.913
	BCAT	12/255	**0.938**	**0.932**	**0.927**	0.922
	BCAT+	12/255	**0.938**	**0.932**	**0.927**	**0.923**

**Table 7:** Ablation study on data augmentation.

Dataset	Defense	w/o	0	2/255	4/255	6/255	8/255
CIFAR-10	BCAT	with	0.863	0.814	0.752	0.682	0.617
		without	0.839	0.763	0.678	0.582	0.500
	BCAT+	with	0.863	0.817	0.761	0.691	0.628
		without	0.841	0.764	0.680	0.583	0.506
	standard	with	0.863	0.809	0.739	0.666	0.602
		without	0.802	0.727	0.653	0.595	0.557
CIFAR-100	BCAT	with	0.675	0.612	0.548	0.474	0.411
		without	0.613	0.520	0.435	0.352	0.292
	BCAT+	with	0.678	0.614	0.544	0.480	0.418
		without	0.620	0.531	0.437	0.355	0.298
	standard	with	0.644	0.565	0.488	0.427	0.395
		without	0.570	0.491	0.419	0.363	0.325

## Data Availability

Publicly available datasets were analyzed in this study. These data can be found in https://www.cs.toronto.edu/~kriz/cifar.html, accessed on 17 March 2023 and http://ufldl.stanford.edu/housenumbers, accessed on 17 March 2023.

## References

[1] Krizhevsky A., Sutskever I., Hinton G.E. (2017). ImageNet classification with deep convolutional neural networks. Commun. ACM.

[2] Redmon J., Divvala S., Girshick R., Farhadi A. You only look once: Unified, real-time object detection. Proceedings of the IEEE Conference on Computer Vision and Pattern Recognition.

[3] Long J., Shelhamer E., Darrell T. Fully convolutional networks for semantic segmentation. Proceedings of the IEEE Conference on Computer Vision and Pattern Recognition.

[4] Szegedy C., Zaremba W., Sutskever I., Bruna J., Erhan D., Goodfellow I., Fergus R. Intriguing properties of neural networks. Proceedings of the 2nd International Conference on Learning Representations.

[5] Goodfellow I.J., Shlens J., Szegedy C. Explaining and harnessing adversarial examples. Proceedings of the 3rd International Conference on Learning Representations.

[6] Eykholt K., Evtimov I., Fernandes E., Li B., Rahmati A., Xiao C., Prakash A., Kohno T., Song D. Robust physical-world attacks on deep learning visual classification. Proceedings of the IEEE Conference on Computer Vision and Pattern Recognition.

[7] Xu K., Zhang G., Liu S., Fan Q., Sun M., Chen H., Chen P.-Y., Wang Y., Lin X. Adversarial t-shirt! evading person detectors in a physical world. Proceedings of the European Conference on Computer Vision.

[8] Li Z., Dong M., Wen S., Hu X., Zhou P., Zeng Z. (2019). CLU-CNNs: Object detection for medical images. Neurocomputing.

[9] Madry A., Makelov A., Schmidt L., Tsipras D., Vladu A. Towards Deep Learning Models Resistant to Adversarial Attacks. Proceedings of the 6th International Conference on Learning Representations.

[10] Kurakin A., Goodfellow I., Bengio S. Adversarial examples in the physical world. Proceedings of the 5th International Conference on Learning Representations.

[11] Papernot N., McDaniel P., Jha S., Fredrikson M., Celik Z.B., Swami A. The limitations of deep learning in adversarial settings. Proceedings of the 2016 IEEE European Symposium on Security and Privacy (EuroS&P).

[12] Su J., Vargas D.V., Sakurai K. (2019). One pixel attack for fooling deep neural networks. IEEE Trans. Evol. Comput..

[13] Carlini N., Wagner D. Towards evaluating the robustness of neural networks. Proceedings of the 2017 IEEE Symposium on Security and Privacy (SP).

[14] Moosavi-Dezfooli S.-M., Fawzi A., Frossard P. Deepfool: A simple and accurate method to fool deep neural networks. Proceedings of the IEEE Conference on Computer Vision and Pattern Recognition.

[15] Chen P.-Y., Sharma Y., Zhang H., Yi J., Hsieh C.-J. Ead: Elastic-net attacks to deep neural networks via adversarial examples. Proceedings of the AAAI Conference on Artificial Intelligence.

[16] Papernot N., McDaniel P., Goodfellow I., Jha S., Celik Z.B., Swami A. Practical black-box attacks against machine learning. Proceedings of the 2017 ACM on Asia Conference on Computer and Communications Security.

[17] Xu W., Evans D., Qi Y. Feature squeezing: Detecting adversarial examples in deep neural networks. Proceedings of the 25th Annual Network and Distributed System Security Symposium.

[18] Dhillon G.S., Azizzadenesheli K., Lipton Z.C., Bernstein J., Kossaifi J., Khanna A., Anandkumar A. Stochastic activation pruning for robust adversarial defense. Proceedings of the 6th International Conference on Learning Representations.

[19] Gu S., Rigazio L. Towards deep neural network architectures robust to adversarial examples. Proceedings of the 3rd International Conference on Learning Representations.

[20] Samangouei P., Kabkab M., Chellappa R. Defense-gan: Protecting classifiers against adversarial attacks using generative models. Proceedings of the 6th International Conference on Learning Representations.

[21] Song Y., Kim T., Nowozin S., Ermon S., Kushman N. Pixeldefend: Leveraging generative models to understand and defend against adversarial examples. Proceedings of the 6th International Conference on Learning Representations.

[22] Meng D., Chen H. Magnet: A two-pronged defense against adversarial examples. Proceedings of the 2017 ACM SIGSAC Conference on Computer and Communications Security.

[23] Athalye A., Carlini N., Wagner D. Obfuscated gradients give a false sense of security: Circumventing defenses to adversarial examples. Proceedings of the International Conference on Machine Learning.

[24] Carlini N., Wagner D. (2017). Magnet and “efficient defenses against adversarial attacks” are not robust to adversarial examples. arXiv.

[25] Schmidt L., Santurkar S., Tsipras D., Talwar K., Mądry A. Adversarially robust generalization requires more data. Proceedings of the Advances in Neural Information Processing Systems 31: Annual Conference on Neural Information Processing Systems.

[26] Krogh A., Hertz J.A. A simple weight decay can improve generalization. Proceedings of the 4th International Conference on Neural Information Processing Systems.

[27] Srivastava N., Hinton G., Krizhevsky A., Sutskever I., Salakhutdinov R. (2014). Dropout: A simple way to prevent neural networks from overfitting. J. Mach. Learn. Res..

[28] Simonyan K., Zisserman A. Very Deep Convolutional Networks for Large-Scale Image Recognition. Proceedings of the 3rd International Conference on Learning Representations.

[29] Sandfort V., Yan K., Pickhardt P.J., Summers R.M. (2019). Data augmentation using generative adversarial networks (CycleGAN) to improve generalizability in CT segmentation tasks. Sci. Rep..

[30] Tokozume Y., Ushiku Y., Harada T. Learning from Between-class Examples for Deep Sound Recognition. Proceedings of the 6th International Conference on Learning Representations.

[31] Tokozume Y., Ushiku Y., Harada T. Between-class learning for image classification. Proceedings of the IEEE Conference on Computer Vision and Pattern Recognition, Computer Vision Foundation/IEEE Computer Society.

[32] Kannan H., Kurakin A., Goodfellow I. (2018). Adversarial logit pairing. arXiv.

[33] Zhang H., Yu Y., Jiao J., Xing E.P., Ghaoui L.E., Jordan M.I. Theoretically Principled Trade-off between Robustness and Accuracy. Proceedings of the 36th International Conference on Machine Learning.

[34] Mao C., Zhong Z., Yang J., Vondrick C., Ray B. Metric Learning for Adversarial Robustness. Proceedings of the Advances in Neural Information Processing Systems 32: Annual Conference on Neural Information Processing Systems.

[35] Li P., Yi J., Zhou B., Zhang L. Improving the Robustness of Deep Neural Networks via Adversarial Training with Triplet Loss. Proceedings of the Twenty-Eighth International Joint Conference on Artificial Intelligence.

[36] Zhang H., Wang J. (2019). Defense against adversarial attacks using feature scattering-based adversarial training. Adv. Neural Inf. Process. Syst..

[37] Yu Y., Gao X., Xu C.-Z. LAFEAT: Piercing Through Adversarial Defenses with Latent Features. Proceedings of the IEEE/CVF Conference on Computer Vision and Pattern Recognition, Computer Vision Foundation.

[38] Chen K., Chen Y., Zhou H., Mao X., Li Y., He Y., Xue H., Zhang W., Yu N. Self-supervised adversarial training. Proceedings of the ICASSP 2020—2020 IEEE International Conference on Acoustics, Speech and Signal Processing (ICASSP).

[39] Liu X., Li Y., Wu C., Hsieh C.-J. Adv-BNN: Improved Adversarial Defense through Robust Bayesian Neural Network. Proceedings of the 7th International Conference on Learning Representations.

[40] Wang Y., Ma X., Bailey J., Yi J., Zhou B., Gu Q. On the Convergence and Robustness of Adversarial Training. Proceedings of the 36th International Conference on Machine Learning.

[41] Rice L., Wong E., Kolter Z. Overfitting in adversarially robust deep learning. Proceedings of the 37th International Conference on Machine Learning.

[42] Yu X., Smedemark-Margulies N., Aeron S., Koike-Akino T., Moulin P., Brand M., Parsons K., Wang Y. (2023). Improving adversarial robustness by learning shared information. Pattern Recognit..

[43] Zhang J., Xu X., Han B., Niu G., Cui L., Sugiyama M., Kankanhalli M. Attacks which do not kill training make adversarial learning stronger. Proceedings of the 37th International Conference on Machine Learning.

[44] Ye S., Xu K., Liu S., Cheng H., Lambrechts J.-H., Zhang H., Zhou A., Ma K., Wang Y., Lin X. Adversarial Robustness vs. Model Compression, or Both?. Proceedings of the IEEE/CVF International Conference on Computer Vision.

[45] Sehwag V., Wang S., Mittal P., Jana S. HYDRA: Pruning Adversarially Robust Neural Networks. Proceedings of the Advances in Neural Information Processing Systems 33: Annual Conference on Neural Information Processing Systems.

[46] Shafahi A., Najibi M., Ghiasi A., Xu Z., Dickerson J.P., Studer C., Davis L.S., Taylor G., Goldstein T. Adversarial training for free!. Proceedings of the Advances in Neural Information Processing Systems 32: Annual Conference on Neural Information Processing Systems 2019.

[47] Zhang D., Zhang T., Lu Y., Zhu Z., Dong B. You Only Propagate Once: Accelerating Adversarial Training via Maximal Principle. Proceedings of the Advances in Neural Information Processing Systems 32: Annual Conference on Neural Information Processing Systems.

[48] Wong E., Rice L., Kolter J.Z. Fast is better than free: Revisiting adversarial training. Proceedings of the 8th International Conference on Learning Representations.

[49] Vivek B., Babu R.V. Single-step adversarial training with dropout scheduling. Proceedings of the 2020 IEEE/CVF Conference on Computer Vision and Pattern Recognition (CVPR), Computer Vision Foundation.

[50] Wu T., Liu Z., Huang Q., Wang Y., Lin D. Adversarial Robustness under Long-Tailed Distribution. Proceedings of the IEEE/CVF Conference on Computer Vision and Pattern Recognition, Computer Vision Foundation.

[51] Ng A.Y. Feature selection, L 1 vs. L 2 regularization, and rotational invariance. Proceedings of the Twenty-First International Conference on Machine Learning, Association for Computing Machinery.

[52] Xu Y., Zhong Z., Yang J., You J., Zhang D. (2016). A new discriminative sparse representation method for robust face recognition via L2 regularization. IEEE Trans. Neural Netw. Learn. Syst..

[53] Caruana R., Lawrence S., Giles L. (2000). Overfitting in neural nets: Backpropagation, conjugate gradient, and early stopping. Adv. Neural Inf. Process. Syst..

[54] Chen T., Kornblith S., Swersky K., Norouzi M., Hinton G.E. Big Self-Supervised Models are Strong Semi-Supervised Learners. Proceedings of the Advances in Neural Information Processing Systems 33: Annual Conference on Neural Information Processing Systems.

[55] Ndirango A., Lee T. (2019). Generalization in multitask deep neural classifiers: A statistical physics approach. Advances in Neural Information Processing Systems 32, Proceedings of the Annual Conference on Neural Information Processing Systems, Vancouver, BC, Canada, 8–14 December 2019.

[56] Noh H., You T., Mun J., Han B. Regularizing Deep Neural Networks by Noise: Its Interpretation and Optimization. Proceedings of the Advances in Neural Information Processing Systems 30: Annual Conference on Neural Information Processing Systems.

[57] He K., Zhang X., Ren S., Sun J. Deep residual learning for image recognition. Proceedings of the IEEE Conference on Computer Vision and Pattern Recognition.

[58] Pang T., Yang X., Dong Y., Su H., Zhu J. Bag of Tricks for Adversarial Training. Proceedings of the 9th International Conference on Learning Representations.

[59] Göpfert C., Göpfert J.P., Hammer B. Adversarial Robustness Curves. Proceedings of the Joint European Conference on Machine Learning and Knowledge Discovery in Databases.

